# Minimising the impact of infectious outbreaks on resident quality of life: A qualitative proof of concept study using the Adult Social Care Outcomes Toolkit (ASCOT)

**DOI:** 10.1371/journal.pone.0316424

**Published:** 2025-09-25

**Authors:** Diane Bunn, Wenjing Zhang, Nick Smith, Oluseyi Jimoh, Jane Greenstock, Mohammud Edoo, Ann-Marie Towers

**Affiliations:** 1 School of Health Sciences, University of East Anglia, Norwich, Norfolk, United Kingdom; 2 Health and Social Care Workforce Research Unit, King’s College London, London, United Kingdom; 3 Centre for Health Services Studies (CHSS), University of Kent, Canterbury, United Kingdom; 4 NHS Midlands and Lancashire Commissioning Support Unit, The Strategy Unit, Birmingham, United Kingdom; 5 UK Health Security Agency East of England Region, Harlow, Essex, United Kingdom; University of California San Diego Division of Biological Sciences, UNITED STATES OF AMERICA

## Abstract

**Introduction:**

Infection control measures (ICMs) used to mitigate the effects of infectious outbreaks in care homes impact on resident quality of life (QoL). This qualitative proof-of-concept study explored whether the Adult Social Care Outcomes Toolkit (ASCOT) could feasibly support care home staff in recognising and minimising these impacts.

**Methods:**

There were two phases involving online interviews with six care home managers/deputies from five homes who had managed notifiable outbreak(s) in the previous six months in two regions of England. Phase 1, using an incident analysis approach, explored the impact of outbreaks and ICMs on resident QoL, mapping data to the eight ASCOT domains. Phase 2 assessed the usefulness of using ASCOT to identify, monitor and minimise impacts on resident QoL during infectious outbreaks. Follow-up interviews were conducted with four care homes from phase 1 and six healthcare professionals with ICM responsibilities. Online interviews were analysed using framework analysis.

**Findings:**

Phase 1: three types of outbreaks (COVID-19, norovirus, chest infections) were discussed. All were managed using standard ICMs: isolation, increased cleaning, and staff wearing personal protection equipment. The impacts of these measures on resident QoL were described. Phase 2: two overarching themes identified: (i) ICMs as a personal cost for the greater good and (ii) the potential of ASCOT in minimising impacts of infectious outbreaks on resident QoL as a tool to support care planning and mitigating impacts.

**Conclusion:**

ASCOT can support planning to mitigate the effects of ICMs for infectious outbreaks on resident QoL.

## Introduction

There are around 17,000 care homes for adults in England, providing 24-hour onsite care for vulnerable people who are unable to be cared for at home. Approximately 5000 cater for those living with a learning disability [1,2]. Care homes are part of a quasi-market, with the majority run by private for-profit and not-for-profit providers. Whilst some care homes have been purpose built, many are adaptations of existing housing stock and residents often live in close proximity, sharing communal areas and sometimes bathrooms. Sizes vary (range 1–250), but the average is 30 beds. Unlike clinical settings, care homes are ‘homes’ first, with rooms containing personal possessions and soft furnishings. Mobility allowing, residents move freely around the home or ‘unit’ in which they live. This home-like environment makes the implementation and impact of infection control measures (ICMs) particularly challenging [3,4] – an issue highlighted during the COVID-19 pandemic when guidance around implementing national guidelines for ICMs was frequently adapted for local situations [5,6].

The SARS-CoV-2 virus has been the major cause of infectious outbreaks in recent years, but it is far from being the only source. Care homes have always had to contend with other infectious diseases, such as influenza and other respiratory infections, gastro-intestinal conditions and scabies, due to the close proximity of residents and staff, and vulnerability of residents. Many diseases are notifiable, requiring care homes to report outbreaks to the United Kingdom Health Security Agency (UKHSA) so that appropriate support from local Health Protection Teams (HPTs) and other community partners can be instituted [7].

Much research has been undertaken into disease-specific prevention, admission, spread, containment and subsequent management of infectious outbreaks [8–10], and care homes have access to a number of guidelines, which cover generic and disease-specific recommendations [11–13]. However, these often involve substantial changes to routines and practices. For example, containment of the SARS-CoV-2 virus meant the introduction of a whole range of ICMs, including: closure of care homes to anyone except ‘essential’ visitors, isolating residents in their rooms, restrictions in movement and activities (for staff, residents and visitors), and staff wearing personal protective equipment (PPE) [14]. The consequences of these ICMs and the impact on the quality of life (QoL) of those living and working in care homes have been highlighted as a cause for concern and an area needing further consideration and support [15,16]. A balance is needed between implementing ICMs to protect against infection and ensuring that unintended consequences on QoL are minimised [16–19].

Resident QoL data is not routinely collected and recorded in the UK [20,21]. In care homes, most residents cannot self-report due to frailty and cognitive impairment [22,23], meaning that alternative methods of data collection, such as through proxies, are required to avoid substantial missing data. Recent research piloting a Minimum Data Set in care homes for older adults in England [22,24] has provided support for the feasibility and psychometric properties of three standardised QoL measures, when completed by staff proxy: the Adult Social Care Outcomes Toolkit (ASCOT)-Proxy [25], the ICEpop CAPability measure for Older people (ICECAP-O) [26] and the EuroQol-5 Dimensions (EQ-5D) [27,28]. The measures capture different underlying constructs (social care-related QoL, capability wellbeing for older people and health-related QoL, respectively) and decisions regarding which is most appropriate to use will depend on the purpose of data collection [22,29].

Exploring the impact of ICMs on resident QoL requires a measure that is sensitive to the impact of social care services (i.e.,: will be able to detect the impact of changes in practice on resident QoL), has been validated in care homes [22,24,30] and with different social care client groups (not only older people) [31,32]. The ASCOT meets all of these requirements. However, it has never been used to explore the impact of ICMs specifically.

### Purpose

In this qualitative proof-of concept study, we explored how resident QoL had been affected during an infectious outbreak, and whether the ASCOT tool had the potential to support care staff in identifying and mitigating effects.

## Methods

This study was approved by University of East Anglia Faculty of Medicine and Health Sciences Research Ethics Committee (reference: ETH2324−1074). There were two phases: Phase 1 explored the impact of outbreaks and ICMs on resident QoL and data mapped to the ASCOT domains of QoL. Phase 2 assessed the usefulness of using ASCOT to identify, monitor and minimise impacts on resident QoL during infectious outbreaks.

### Participants and recruitment

Phase 1 interviews were conducted with six care home managers/deputies (CHMs) from five care homes providing care for adults in the east and south-east of England. All had managed notifiable outbreak(s) in the previous six months. In Phase 2, we conducted four follow-up interviews with the same CHMs (although two were unable to participate within the study period) as well as six healthcare professionals (HCPs) involved in infection prevention and control in care homes. All those providing informed consent (written, submitted via an online form) were interviewed.

Study information was disseminated via ENRICH (Enabling Research in Care Homes, Phase 1 only), newsletters, social media, UKHSA, and contacts within infection control teams. If interested, potential participants were invited to contact the research team or access our study website [Adult Social Care Outcomes Toolkit (ASCOT)] for full study information and to record consent. In both phases interviews were conducted by experienced care-homes’ researchers (OJ, NS, WZ) online (mean 45 minutes), recorded and transcribed using Microsoft Teams. Transcriptions were not returned, but participants had access to the recording. Excel spreadsheets facilitated data management and analysis and participants were allocated a unique identification number prior to analysis. Care home managers were offered £100 shopping tokens for each interview.

### Phase 1 (February-April 2024): Mapping impacts of infectious outbreak on resident QoL

Following an incident analysis approach [33,34] and using a pre-prepared Topic Guide (S1 File), we explored:

1. What happened?
2. What actions were taken?
3. Possible impacts on resident QoL and how these mapped to the eight ASCOT domains of care-related QoL: control over daily life, occupation, social participation, personal safety, personal comfort and cleanliness, home comfort and cleanliness, food and drink, dignity.
4. What could have been done differently to: (i) minimise any negative effects; (ii) improve on existing good practice?

Initially, salient points and key findings were mapped to the ASCOT domains, a process duplicated independently by research team members. Within the team, we discussed theoretical possibilities of where ASCOT may have been helpful in supporting resident QoL during an infectious outbreak. This preliminary analysis informed the Topic Guide and vignette (S2 File) for Phase 2 interviews.

### Phase 2 (April–May 2024): Exploring potential of ASCOT in minimising impacts of infectious outbreaks on resident QoL

Interview discussions, guided by the vignette (provided in advance) and Topic Guide, explored views on the usefulness of using ASCOT to identify, monitor and minimise impacts on resident QoL during infectious outbreaks. We used Framework Analysis to analyse the data by participants and ASCOT domains, following six stages: familiarisation, coding, analytical framework development and application, indexing, charting, interpretation [35,36]. We labelled data segments (coding) in line with the topic guide. Through familiarisation with the data and analytic discussions, a theoretical framework was developed and applied to the data, which were then indexed and charted, as per the framework. Themes were identified, refined and interpreted in relation to the study purpose and research question.

### Public involvement

A family carer of someone who lived in a care home for older adults during the COVID-19 pandemic was involved in shaping the design of this research and analytic discussions, attending research team meetings to share their own experiences of the impact of ICMs and the relevance of this research for care home residents, staff and family members. Two care home managers, including one who is a family carer, provided valuable feedback on participant recruitment materials and supported recruitment activities.

This study report follows COREQ guidelines [37], S3 File.

## Findings

### Phase 1: Incident analysis

All six CHMs, representing five care homes, had extensive experience in care (15 + years), and had worked their way up from carer to current positions. Four care homes provided care for older adults (>65 years), including those living with dementia, and one for younger adults (18–65 years) living with learning disabilities. Two homes discussed two separate outbreaks. Outbreaks discussed were COVID-19, chest infections, norovirus (Table 1). Inevitably, due to the recentness of the COVID-19 pandemic, many discussed their current experiences in relation to that.

**Table 1:** Care home outbreak information.

Care home No.	01	02	03	04	05
**Care provision**	Younger adults living with learning disabilities	Older adults, including those living with dementia			
**Capacity**	7 across two units	20-30	40-50	30-40	70+
**Outbreak(s) discussed**	COVID-19 COVID-19	Norovirus	COVID-19 Chest infection	Norovirus	Chest infection
**Approx duration of outbreak**	14 days 16 days	11 days	COVID-19: 10 days Chest infection: 4 weeks	8 days	12 days
**Approx no. residents affected**	2 2	5	COVID-19: 14 Chest infection: 8	23	6
**Approx no. staff affected**	1 4	3	COVID-19: 4 Chest infection: N/K	30	0

NB: “Chest” infection(s) was the term used by interviewees. These would generally be referred to by health protection/public health professionals as “respiratory” infections [38].

1.1 **What happened?**

All participants were unsure how the infection entered the home. From the three types of infectious outbreaks described to us, norovirus and COVID-19 seemed to have the most impact (on both residents and staff). Chest infections had the least impact in the home as a whole, possibly because fewer staff tend to contract chest infections, so staffing levels are not impacted to the same extent. There also seemed to be less concern about implementing extensive ICMs during these outbreaks, compared with norovirus and COVID-19. In all the outbreaks discussed, the impact on the home increased when duration was prolonged and/or there were greater numbers of residents and staff effected. In one care home that had norovirus, there was a substantial impact, where 30 staff were affected, as well as the majority of residents. Numbers of staff infected in other outbreaks seemed less significant, although some participants discussed how testing for some conditions is not required, and staff may continue to work if asymptomatic, or have milder symptoms.

1.2 **Actions taken**

**Fig 1:**
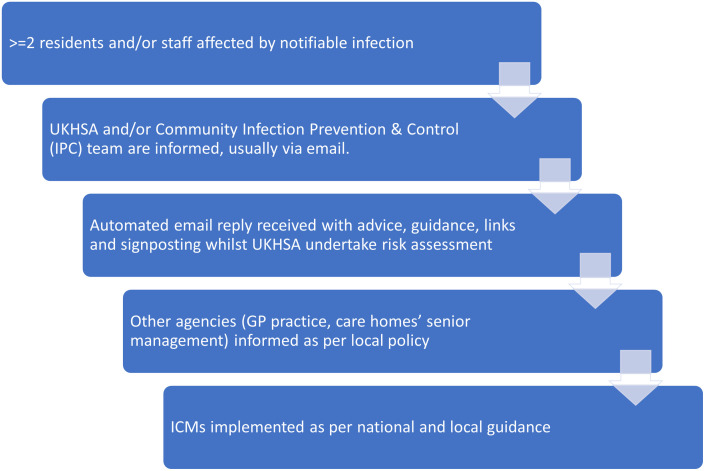
Standard Actions Taken by Care Home Managers when there is an Infectious Outbreak.

All interviewees told us that they followed standard guidance (Fig 1), and there was a sense of ‘we know this, we are on familiar ground’ due to their experience, particularly COVID-19. The key priority was keeping residents safe from both transmitting and contracting the infection.

1.3 **Impacts on residents’ QoL, mapped to ASCOT’s eight domains**

Whilst the focus of this study was to explore the direct impacts of infectious outbreaks on residents’ QoL, as identified by care home managers/deputies, we also explored impacts on staff, knowing that these would have consequential effects on residents.

Using the ASCOT domains, we identified that resident QoL was likely impacted on each domain (Table 2). Restricted movements, zoning and isolation in, and of itself, were the major underpinning cause. Impacts were wide-ranging, resulting in reduced control over daily life, reduced social participation and occupation, changes in food and drink provision, and changes in support with personal cleanliness, all associated with reduced choice. Staff recognised that residents’ Dignity (the impact of support and care on how residents feel) was also negatively impacted. The consequences of ensuring residents were safe from infection (Safety) and residing in a clean home (Accommodation Cleanliness and Comfort), especially during norovirus outbreaks, were that other safety issues, such as the impact on mental health and close monitoring of those with dysphagia, were deprioritised. One participant reported an increase in behaviour that challenges amongst residents. All participants said that ensuring a safe environment for people who liked to walk freely around the home was a major challenge.

**Table 2:** Examples of how infectious outbreaks impact on resident QoL, mapped to ASCOT’s domains.

	Direct Impacts of all ICMs on Resident QoL					
ASCOT Domains	Isolation, zoning, restricted movements around home	Implementing other ICMs	Changes in care needs	Changes in CH routines	Residents with impaired understanding and/or increased anxiety	Quotes
**Control over daily life**	Restrictions on movements & activities.	Expected adherence to ICMs.	Compliance with testing & other care related to the infection. Some testing (e.g., nasal swabs) is unpleasant.	Changes in daily routines imposed. Lack of communication between staff about changes with residents.	Increased anxiety due to decreased control.	*‘They felt very isolated and felt very not in control of their lives basically.’* *(04, CHM)*
**Occupation**	Reduced activities & stimulation, including organised activities. More reliance on passive, solo activities such as watching TV. Boredom.	Any activities restricted to using disposable or washable products. Location of activities often restricted to residents’ rooms.	Changes in type of activities residents wish to participate in, depending on their condition.	Fewer or no groups activities. Increase in 1-2-1 activities.	Unable to move around the home and/or leave the home at will. Short concentration span creates issues.	*“That was spending time with them, not just going in, getting them ready, getting them dressed, getting them do whatever they needed. It was about having that special one to one time with them because they are stuck in their room.”* *(03, CHM)*
**Social participation**	Less ‘bustle’. Reduced staff/resident interactions & family contacts. Reduction in social activities.	Mask wearing impedes communications & may cause anxiety. Staff not eating with residents.	Greater reliance on digital communication with families.	Less ‘bustle’. Increased use of agency and unfamiliar staff mean that content of interactions & topics of conversations change.	Reduced social participation liked by some, who find socialising hard.	*“So many of them rely so much on a social element.”* *(01, CHM)* *“It’s a massive impact and then they couldn’t see their families either’. (04, CHM)*
**Personal safety**	Fewer falls.	Masks & other PPE worn by staff causes distress and fear.	Less monitoring for some issues, e.g., dysphagia. Reduced contact with visiting HPs, and delays in care, e.g., routine blood tests.	Increased monitoring for other issues, such as fluid intake. Unfamiliar staff don’t know residents & their needs.	Increase in self-harming and adverse behaviours. Masks & other PPE worn by staff causes distress and fear.	*“Because they’re not well, you’re checking on them more” (02, CHM)* *“…. there was a lot more PPE […] I think that was the only fear for them, in that they weren’t used to that, being in that sort of environment.”* *(05, CHM)*
**Personal comfort and cleanliness**	Prioritising of hygiene needs, e.g., beds not made/changed if not soiled	Bathing & showering replaced by washes in rooms, to prevent cross-contamination when using shared spaces.	Increased where needed (e.g., diarrhoea & vomiting)	Unfamiliar staff don’t know residents, their needs and preferences.	Reduced monitoring of how residents maintain personal hygiene if usually semi-independent.	*“They couldn’t have baths and showers because it’s cross contamination, but they still got full body washes twice a day, minimum. Unless they are incontinent obviously, but definitely twice a day they would still get full personal care”.* *(03, CHM)*
**Home comfort & cleanliness**	Laundry bags & other clinical waste kept in rooms until disposal.	Increased clutter in corridors with extra bins, sanitation stations etc. Deep cleans after outbreak leaving strong smell.	Increased cleaning.	Prioritising, e.g., beds not made. Increased cleaning. Odours of bleach, diarrhoea & vomiting.	Coping with all the changes.	*“Bleach. Hand gels, washing places are set up everywhere just to make it safer for the staff as well as the residents”* *(03, CHM)*
**Food and drink**	Diminished dining experience. Food & drink served in rooms. Reduced choice & autonomy in providing for self.	Staff no longer sit with residents at mealtimes. Perspex screens at tables.	Lack of, or changes in, appetite and preferences. Providing appropriate foods & drink for those with diarrhoea & vomiting. Increased monitoring around fluid intake. Increased support needed with eating & drinking due to decreased abilities.	Food & drink served in rooms. Delays in serving. Unfamiliar staff don’t know residents, their needs and preferences.	Reduced choice & autonomy when providing for self.	*“Massive impact. Residents that had to isolate in their bedrooms [….], they couldn’t come out and join in the activities. They couldn’t come to the dining room, which is very sociable, to have meals with other people.”* *(04, CHM)*
**Dignity**	Staff zoning (i.e., staff allocated to residents), so residents not seeing favourite staff or staff they feel comfortable with.	Bathing & showering replaced by washes in rooms, to prevent cross-contamination.	Requiring additional help/not as much help as needed or liked. Ensuring residents with diarrhoea & vomiting are supported in a manner which respects their dignity	Unfamiliar staff who don’t know residents and their care preferences and needs. Having washes in rooms, instead of showers/baths.	Dislike of COVID-19 testing (nasal and throat swabs)	*“But you really need to make sure the people are isolated because of their dignity as well, because they don’t want to feel unwell in front of people, especially if you’re vomiting”.* *(02, CHM)*

Glossary: Care Home Manager = CHM.

Interviewees discussed that the increase in workloads and staff shortages (due to sickness and relocation of vulnerable staff) during an outbreak, meant that all work was triaged, and priorities identified, so that some work was left undone, modified or reduced. Inevitably this affected residents’ QoL. Some examples provided were that whilst staff prioritised cleaning and laundry, beds may not be made; and whilst food and drink continued to be served, there may be reduced choices as to what was available and when, and meals may be served in bedrooms, rather than the social space of the dining room.

Overall, changes in routines and the surrounding uncertainty could be upsetting for many residents.

To offset staff shortages, some interviewees discussed employing agency staff or relocating staff from other homes in the group. This was associated with additional negative impacts on many domains of residents’ QoL, due to unfamiliar staff not knowing the layout of the homes, procedures, routines, or residents and their care needs. There were also additional infection risks and increased financial costs. Interviewees reported that many residents found that staff wearing masks increased resident anxiety due to difficulties with recognition and communication. Additional staff workloads were associated with increased resident care needs (due to illness), extra documentation, learning, training, implementing and monitoring ICMs, and communicating with external agencies and families regarding the outbreak. Where there was conflicting ICM advice, this further added to workloads. Some interviewees talked about reduced healthcare visits from external HPs, attending to essential care only, and the impact this had on the residents’ health. However, all participants described how roles merged, with a flattening of hierarchies as everyone worked together. A participant, who managed a group of smaller residential units for adults living with learning disabilities, questioned the equity of blanket ICMs for those living in care homes, compared to those living in their own homes.

1.4 **What could have been done differently to (i) minimise any negative effects; (ii) improve on existing good practice?**

Regarding what would help minimise impacts on resident QoL, interviewees emphasised that effective planning was essential, including risk assessments for both residents and staff, having guidance in place, and ensuring sufficient stocks of PPE and cleansers etc. They also noted that more and quicker support from external agencies, along with better communication between these agencies, would be beneficial, as external support tended to focus primarily on procedural issues. All interviewees stressed the benefits of a strong team within the care homes, with a culture of supporting each other, as the demands of an outbreak were physically and mentally exhausting. Additional support came from ‘small acts of kindness’ (e.g., ordering in ‘take-aways’), ‘open-door policy’, management ‘working on the floor’, informal debriefing and reflecting together. More formal debriefing sessions and access to counselling were mentioned by some. However, there was no mention of whether any debriefing, reflecting or counselling were available to residents. Whilst recognising the impact of an outbreak on mental health of residents, including their fear of becoming ill with the infection, it was not discussed how this was managed. One interviewee thought that there should be resources available to explain what an outbreak was and how and why it was being managed the way it was, for people with cognitive impairments. Some interviewees discussed the creative approaches to supporting occupation and social participation during the COVID-19 pandemic, due to its prolonged nature, which could be explored further and adapted for shorter outbreaks when residents are being isolated.

### Phase 2: Exploring the potential of ASCOT in minimising impacts of infectious outbreaks on resident QoL

The vignette resonated with all interviewees, and supported the development of a focused discussion. Interviewees felt that many of the issues described were not unusual, albeit not always representative of good practice, and were keen to provide ideas of how deficiencies in care could be addressed. At a later stage in the interviews, interviewees were asked to consider whether the ASCOT tool would be supportive in helping to frame their concerns and care approaches, with all interviewees agreeing that this would be useful.

We found a remarkable consistency of views across all participants, although differences were noted between CHMs and HCPs regarding the role of HCPs in preparing and supporting care homes for infectious outbreaks.

Two themes were identified:

1. ICMs: a personal cost for the greater good
2. Potential of ASCOT in minimising impacts of infectious outbreaks on resident QoL
3. ***Theme 1: ICMs: a personal cost for the ‘greater good’***

Theme 1, about the impact of ICMs on resident QoL, resonates closely with Phase 1 findings. With the addition of HCPs, who echoed the views of CHMs, this further confirmed our findings. All interviewees recognised that the aim of ICMs is to protect the community as a whole whilst supporting and caring for the individual, but acknowledging that implementing ICMs impacted on the QoL of residents, as well as their family and friends and care home staff. Isolation was seen as the main ICM, and the one having the most detrimental impact on resident QoL, thus dominating interview discussions.

*“…obviously we have to isolate in order to reduce the impacts on the wider care home and [consider] health and wellbeing the longer an outbreak goes on.”*

*(08, HCP)*

Whilst the difficulties of implementing isolation were acknowledged, as well as the consequent impact on QoL, isolation as a means of infection control was not questioned, but accepted as the norm. However, in Phase 2, participants (both CHMs and HCPs) seemed to acknowledge that ‘isolation’ could be interpreted in different ways:

*“Sadly, we are a predominantly dementia home so that it [isolation] is not always possible.”* (*03, CHM)*

*“I just don’t think there’s recognition that isolation can be done in multiple different ways, and there are things that we can do, while we do isolate someone, to make them more comfortable.”*

*(08, HCP)*

Phase 2 participants’ views on the impact of other ICMs on resident QoL corresponded with those in Phase 1 (Table 1), acknowledging that the combined effect of multiple changes was unsettling for both staff and residents, but especially for those living with dementia and/or a learning disability:

*“It’s a huge upheaval for them, and I think sometimes for staff as well, because it takes them out of their usual routine.”*

*(01, CHM)*

However, as one CHM pointed out, isolation is not necessarily seen as detrimental by everyone, and may actually be enjoyable, particularly for those who find socialising difficult:

*“Demands significantly reduced […] loving life because he could slob on his bed, watch his telly and. have his food and drink delivered at any given point throughout the day. But the majority really struggled with isolation.”*

*01(CHM)*

Essentially, ICMs, and isolation in particular, have an impact on all aspects of resident and staff QoL, but the extent of these is dependent on many factors, including type of virus and its effects, resident’s own situation and views, organisational factors, staff numbers and approaches to care.

***Theme 2: Potential of ASCOT in minimising impacts of infectious outbreaks on resident QoL (preparing for and managing outbreaks)***

As many impacts are preventable or modifiable, appropriate planning and preparation are key in mitigating impacts. The ASCOT tool was seen as being helpful in identifying what particular aspects of resident QoL were likely to be impacted.

Infectious outbreaks in care homes are seen as being inevitable, having a major impact on everyone (residents, staff, families). At the time, managers are overseeing a challenging situation, leading one sympathetic HCP to describe it as:

*“It’s a challenge […] sometimes it can be sort of crisis management, can’t it? […] It’s survival.”*

*(07, HCP)*

Care homes always prepare for infectious outbreaks and implement ICMs as required, guided, and supported by external agencies, such as the UKHSA and local Infection Prevention and Control (IPC) teams. This division of responsibilities was acknowledged by all.

*“We’re at the end of the phone, we can support them, but that’s about as far as it goes. We wouldn’t be going into there, because there’s nothing that we could actually do physically to help them.”*

*(10, HCP)*

*“They give you instructions on how to deal with it, but they don’t give you a lot of support in any other way [...], they just tell you what to do […] like how to stop the virus or whatever spreading. That’s about it, really. I don’t know what they could do, I mean other than provide more PPE.”*

*(02, CHM)*

Whilst there was consensus between CHMs and HCPs on roles and responsibilities, the way advice and guidance were communicated and perceived differed, with one HCP recognising that misunderstandings about which ICMs to use, when were common:

*“Being there to give up-to-date guidance and […] expertise […] what’s appropriate, what isn’t appropriate and what’s too much and what is perhaps not enough […] there’s a lot of misunderstanding.”*

*(07, HCP)*

However, CHMs’ experiences of external HCP support did not always meet their expectations, anticipating that responses would be quicker and more personalised (rather than receiving a standard out of office reply with general advice), and cohesive. Participants commented on the lack of co-ordination between services, and differences in guidance:

*“You have the infection control link meetings that you have to attend, but there’s not really any help when you’re actually in the midst of an outbreak [...] This recent one we’ve just had, we contacted them on the Friday and it was the Tuesday before we got anything back, and that was just an email with guidance [...] They [infection control people] wanted stool samples to confirm what it was, but they hadn’t even told our surgery that our staff members would be bringing them up. So we had loads of staff going up there […] and the doctors wouldn’t take them because they didn’t have reason to test them! So that was a bit of a nightmare. We’ve never had any calls after to see if everything’s over and done with, no emails.”*

*(03, CHM)*

There was universal support for ASCOT to support structured conversations about residents’ QoL between residents, families and care home staff. Participants felt it would be useful to provide personalised information to support resident QoL both as part of routine care and to frame discussions around preferences and needs when preparing for an outbreak.

*“Knowledge is power. I think the more knowledge you can provide anybody, the better outcomes it has for those we are caring for.”*

*(01, CHM)*

This support for ASCOT came with caveats regarding conciseness, maintaining contemporaneousness, adaptability for different kinds of infectious outbreaks, and with the expectation that the tool would inform thinking/planning. Participants did not want a data collection tool to use during an outbreak, where completing it would increase workloads, rather than supporting care:

*“When you are in […] an outbreak, you are bogged down with the very nitty gritty of just daily survival... so if you can plan… perhaps added to the care plan, then it would be useful, because you’re not thinking there and then, because you haven’t got time to think about what would make their QoL better during that time.”*

*(02, CHM)*

Embedding ASCOT into routine care would also mean that staff would be familiar with the domains when considering the impact of ICMs during an outbreak. Several participants discussed providing a one-page ‘isolation care plan’ or ‘grab sheet’, readily available during an outbreak, e.g., on the resident’s door and/or for handover, and that it would be particularly useful for agency staff, although all staff would find it helpful in benefitting resident care. Following the outbreak, it would be useful for debriefing.

Both CHMs and HCPs discussed their views on how ICMs impacted on resident QoL, and participating in this study seemed to lead some HCPs to think that providing advice on supporting resident QoL could become part of their role in the future:

*“It would help us identify if we could do anything better, it could help us identify what we’re doing.”*

*(04, CHM)*

*“I can see us using something like this [ASCOT], saying: how is this person going to be impacted by the isolating?”*

*(08, HCP)*

Although, the value of ASCOT was discussed, one CHM was keen to point out that they already had ‘isolation care plans’ in place which included many of the ASCOT domains, although not formally described as such.

*“That [isolation care plan] come out as soon as there’s kind of any outbreak situation and we’ve got a protocol to alert families and all of those kinds of things... it helps everybody, and staff actually welcome it because they kind of know what to do. They’ve not got to pluck things out of the air. There’s a list of things for them to go and have a look at for what can I do?”*

*(01, CHM)*

## Discussion

### Impact of infectious outbreaks on resident QoL and ASCOT’s use

**Fig 2:**
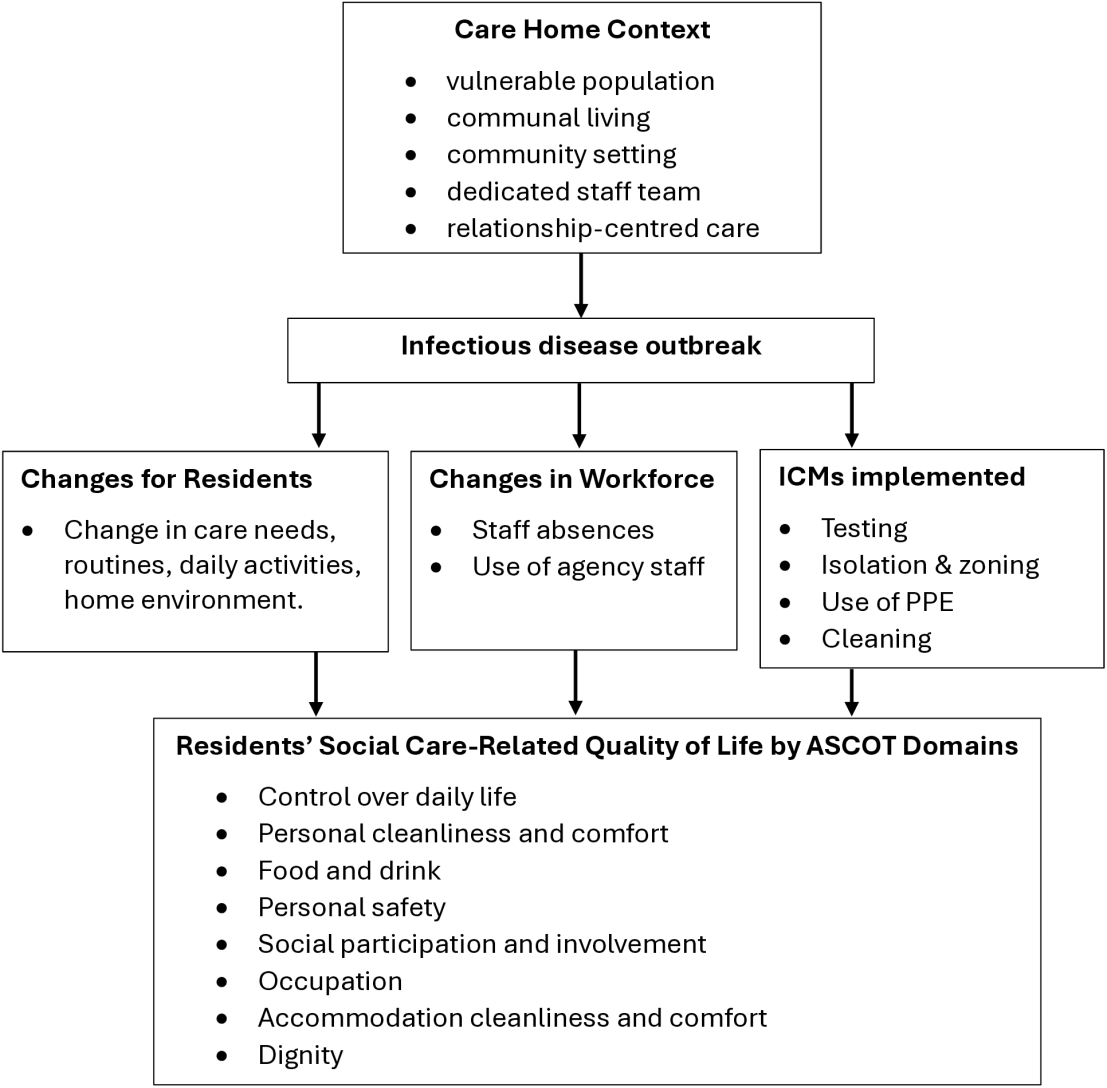
Conceptual model ASCOT and infectious outbreaks.

In this qualitative proof-of-concept study, we explored how resident QoL may be affected during an infectious outbreak, and whether the ASCOT tool had potential to support care staff in identifying and mitigating effects (Fig 2). Impact on resident QoL was well-documented during the COVID-19 pandemic, probably because of its prolonged nature and extensive use of ICMs [19,39], but impacts in shorter, and less widespread infectious outbreaks are less well-known and understood. We have demonstrated that there are likely to be impacts on resident QoL resulting from other infectious outbreaks, but the type and extent of these will vary depending on the infection, due to differences in cause, modes of transmission, effects and management as well as individual resident preferences and local care home systems. Whilst some participants discussed how they already consider QoL and how to mitigate effects of some infectious outbreaks, all agreed that ASCOT seems to provide a more structured approach, and especially one that would support person-centred care.

Developing more person-centred care approaches is a recognised research amongst care home staff and researchers [40]. Improvements in advanced planning for infectious outbreaks was a key issue arising from the COVID-19 pandemic, with recommendations that HCPs should work more closely with care homes to enable this [41–43] with clear, cohesive and non-judgmental support [5,18,44]. Dissonance between HCPs and CHMs perspectives of help and support offered and expected was apparent in our findings. However, both groups discussed the benefit of using ASCOT, indicating the tool might provide a shared language and support conversations between professional groups about mitigating factors in a person-centred way Table 3.

**Table 3:** Potential ASCOT use in outbreak planning and management: key recommendations.

Theme	Recommendation	Purpose/benefit
1. Planning & preparation	Embed ASCOT in care plans pre-outbreak (as routine)	Anticipates QoL needs (and changes) during incidents
	Create brief ASCOT-informed outbreak management plan, e.g., isolation care plan	Quick reference for all staff, including agency workers
	Use ASCOT as a planning checklist	Guides person-centred outbreak preparation
2. Staff support	Train staff in ASCOT use routinely	Builds familiarity & confidence
	Include ASCOT in debriefs post-outbreak	Supports learning, reflective practice and wellbeing. Prepare for future outbreaks.
3. Resident-centred Care	Map ICMs against ASCOT domains to assess impact on individuals.	Minimise ICM impact, e.g., isolation for people with dementia/LD.
	Adapt social and occupational activities during isolation	Maintains engagement and mental wellbeing
	Offer simple explanations of outbreaks to residents with cognitive needs	Reduce anxiety, promote wellbeing
4. Implementation notes	Keep ASCOT-informed tools concise and practical during outbreaks	Avoids overburdening staff
	Do not use ASCOT as a measure during outbreaks	Maintains its use as a planning/support tool, not as an evaluation instrument

### Implementation challenges and care home context

Implementing new practices can be challenging [45], but our participants discussed how implementing ASCOT into routine care practice and using it to plan for infectious outbreaks (perhaps as a checklist) would be beneficial. During outbreaks, staff could then be provided with an accessible one-page summary, to support care during the challenging circumstances of increased resident care needs, staff shortages, and unfamiliar staff. However, interviewees in this study were not in favour of ASCOT being a measure of impact or an assessment of changes in resident QoL, neither of which are intended functions of the ASCOT tool during outbreaks, although ASCOT has been used by others in this way in routine practice [46]. Further work would, therefore, be needed to explore the acceptability and implementation of ASCOT to support care-related QoL during infectious outbreaks, including whether the validated proxy versions would be valid in these circumstances [30].

All care homes in our sample included people living with dementia or learning disabilities and the difficulties of implementing ICMs in these settings, with consequent impacts on resident QoL, was acknowledged by all. Care homes are very different in that they are neither clinical nor domiciliary settings, but are unique in that they provide ‘homely’ long-term care within a community-living context. They require bespoke approaches to identify effective ICMs [14,47–49], which also recognise that there is a balance to be achieved between the rights, comfort and QoL for the individual and the needs of the community [47].

### Study limitations and future research

In this study we used convenience sampling. In to include CHMs from care homes of varying sizes, offering standard residential care as well as for those living with dementia and learning disabilities, and with experience of three common infectious outbreaks. ‘Data saturation’ [50] is often used to indicate when enough data has been collected to draw necessary conclusions, and further data collection is unlikely to provide additional insights. Despite being a small study, and given the remarkable consistency of findings, it is likely that we reached data saturation.

There were no nursing homes in our sample, so transferability of findings to these types of homes, where registered nurses are included in the staff mix, is unknown. In the UK, residents in nursing homes are likely more dependent, requiring higher levels of care, so differences in resident outcomes, including how resident QoL is impacted during infectious outbreaks, could be affected differently [51].

Whilst we were able to explore just three common types of infectious outbreaks [52], we noted distinct differences between them. Norovirus affected many more people (staff and residents) over a relatively short period of time (≈eight days), leaving those affected symptomatic and feeling unwell. In contrast, an outbreak of chest infections seemed to affect residents more than staff, and COVID-19, whilst affecting both staff and residents, staff found it more difficult to manage isolation if residents were asymptomatic and had reduced understanding of why ICMs were necessary. When considering transferability, these findings need to be explored in a larger study which also covers a wider range of infections and includes perspectives from residents, family and those in other caring and support roles (such as other care staff, General Practitioners and Health Protection Teams).

We have only focused on impact on QoL for those already living in care homes, however how routine preventative measures affect QoL when adults first move into care homes remains unknown.

An additional issue for care homes, is managing staff shortages due to increased sickness rates and resident care needs, and how these impact on resident QoL during an infectious outbreak. Whilst staff shortages were discussed by our participants, the related issues of presenteeism and fair recompense for staff who are ill were not discussed in relation to how effective management of this may, in itself, be an effective ICM so that staff do not feel compelled to work when unwell and risk becoming a possible carrier [53].

## Conclusions and further research

In this study, we identified that both the infectious outbreak itself and the ICMs used impacted on resident QoL and that ASCOT could be useful in planning for these events, supporting person-centred care. However, given that each type of infectious outbreak has distinct features, further work is needed to identify what kinds of impacts relate to each infection, which ones may be modifiable and how ASCOT may be applicable in each circumstance. Involving residents, their families and other stakeholders is key. Additionally, understanding how implementation strategies may be effective in supporting change and sustainability is required [54]. Finally, further evaluation of the appropriateness and effectiveness of various ICMs in care homes, particularly isolation and appropriateness for people living with dementia, is urgently needed.

## Supporting information

S1 File Topic Guide (Phase 1). (PDF)

S2 File Vignette & Topic Guide (Phase 2). (PDF)

S3 File COREQ Checklist. (PDF)
