# Supplementary material for: Minimising the impact of infectious outbreaks on resident quality of life: A qualitative proof of concept study using the Adult Social Care Outcomes Toolkit (ASCOT)
Source: PLoS One. 2025 Sep 25;20(9):e0316424. doi: 10.1371/journal.pone.0316424 (PMC12463202; doi:10.1371/journal.pone.0316424)
Supplement: S1 File — (PDF) [file pone.0316424.s001.pdf]

Supplementary File 1: Topic Guide, Phase 1

1. Introduction

- Can you please briefly describe your current role and professional background?
- Could you tell us a little bit about [the care home]
- What responsibilities do you have with regard to supporting residents and staff in the care home?

2. Breakout information & causes

- Can you give me more information about the *[infectious outbreaks – refer to the specific info from UKHSA]* in *[Year/Month]*?
- In your opinion, what were the causes of the outbreak(s) in this care home?
- What prompted you to notify the UKHSA of the outbreak? *(thinking about length of time/numbers of residents/local policy around this)*

3. Management of the outbreaks

- How did you (or the home) respond to the outbreak[s]?
  Can you briefly describe the kinds of infection control measures that you put in place?
  - What support did you provide to the residents due to the outbreak?
  - What support did you provide to the staff due to the outbreak?
  - How did you communicate and support families and friends of the residents, during the outbreak?
- Are there any differences in managing different types of outbreaks [if this care home reported more than one in the past two years]?
- What types of information and data do you collect for an infectious outbreak?
  - Can you tell me more about the way you communicate the incident with the UKHSA and/or local health protection teams?
  - Any other agencies or authorities you need to inform or get involved with?
- Are there any particular challenges or barriers in managing the outbreak?
  - Prompt: daily operation, internal & external communications, reporting & recording

4. Impact on residents, staff & families

- In your view, what were the impacts of the outbreak(s) on the quality of life (QoL) of residents?
  - Prompts: ASCOT domains (personal cleanliness and comfort, food & drink, safety, clean and comfortable accommodation, social participation and involvement, control over daily living, occupation)
  - How did you address these impacts? How did these impact on residents?
- How about the impact on staff and the operation of the home?
  - How did you address these impacts?
  - In your view, did the outbreak or the measures you took to manage it impact on families/visitors? If yes, how?
  - How did you document the impacts and your actions/interventions?
  - Prompts to explore: recorded in individual resident notes, and/or more generally (*either verbally or at care home level in a final report; written/digitally*)?
- Did you share any impacts on resident QoL with external infection control agencies?

5. Looking forward

- What are the key lessons you've learned from managing the outbreaks, that can be used in the future?
- What would help your management and documentation of the outbreaks?
- What could have supported the quality of life for residents, before, during and after the outbreaks?

6. Wrap up

- Do you have anything else to add?
- Is there anything you would like to ask me?
