# Supplementary material for: Minimising the impact of infectious outbreaks on resident quality of life: A qualitative proof of concept study using the Adult Social Care Outcomes Toolkit (ASCOT)
Source: PLoS One. 2025 Sep 25;20(9):e0316424. doi: 10.1371/journal.pone.0316424 (PMC12463202; doi:10.1371/journal.pone.0316424)
Supplement: S2 File — (PDF) [file pone.0316424.s002.pdf]

Supplementary File 2: Vignette & Topic Guide, Phase 2

Quality of life vignette: Barbara

Barbara lives in a care home for older adults. She is 85 years old, has mild dementia, mobility issues, and requires help to wash and dress. The home provides care for 56 residents in a large Edwardian house, which is challenging for some residents to move around unassisted. It is quite homely, though in places is slightly tatty and could do with a bit of decorating. The home is situated in a seaside town and has views looking out over the sea. All residents have their own rooms, but they vary in size and decoration, and in the level of personalisation, but all have basic furniture, a bedside cabinet, a chest of draws, and a wardrobe. Each room has a sink, but only a few have en-suite toilets. There are several resident lounges. Like the rest of the home, they vary in size, are clean and some have stunning sea views.

Barbara has a room on the first floor. The door has a printed sign with Barbara's name and room number, 24, on it. Like most of the rooms, it is not ensuite, but it does have a small sink in the corner. Barbara likes to put her toiletries around the sink, though staff often put them back in the cabinet below the sink. The room is quite plainly decorated and has a few key items of furniture. There are several pictures of Barbara's family and friends in frames both on the wall and on top of the bedside cabinet and the chest of draws. On the chest of draws is an empty vase, usually Barbara's family bring in flowers which they put in the vase. The sink area looks very clean, as does the bed which Barbara is spending a lot of time in right now. The rest of the room, while not dirty, looks like it needs a bit of tidying up. The room, like most of the home, smells of a combination of strong cleaning products, bleach, and, in the background, it is possible to make out the odour of vomit and faeces.

Barbara herself has been spending most of her time in bed. Though recovering, she was one of the first residents in the home to come down with the Norovirus that a large number of residents and staff continue to experience. Still feeling weak after her bout of the virus, she is now able to drink and eat small amounts of food without vomiting and diarrhoea. Next to the bed is a jug of water, and in easy reach, there is also a glass of water that Barbara sips from regularly. Staff have brought her a small plate with two crackers on it. Staff periodically check that Barbara is drinking water safely, and replace the jug with a fresh one, but Barbara would really like a hot cup of tea!

Barbara looks a little pale, but is sitting up in bed, in a clean nightdress, and though her hair has been combed, it doesn't appear to have been washed recently. She looks to be reasonably comfortable, though at regular intervals during the day she asks staff to readjust her pillows. Sometimes she does this when they are delivering drinks, checking on her, or providing personal hygiene, at other times she presses the call button. Sometimes it takes staff a while, but they do eventually come. They are polite and helpful, but do not chat too much. Barbara struggles to work out if she recognises some of the staff, as they all have face masks on to help prevent the transmission of the virus. Barbara says to one that she hasn't met them before. The member of staff explains that it is their first day in the home, she has been sent by an agency to help out while so many of the regular staff are off with norovirus. In a conversation later, Barbara tells you that while the "new girls" seem very nice, she does miss seeing the "regular girls" and Dave, a care worker who always comes and chats with her. She likes to ask him about his dogs.

Unlike some of the residents, Barbara does not have a television in her room. During the morning, she seems to do very little and looks quite bored. She intermittently dozes off to sleep. In the afternoon, a member of staff brings her a magazine to read. They tell Barbara that there is a nice crossword she could do. Barbara picks up the magazine, but promptly puts it down again. Barbara needs to use her reading glasses to read most of the print in the magazine and can only make out the titles of articles and some of the pictures. When

that member of staff comes back to check if Barbara needs a fresh drink, Barbara asks if they can get her reading glasses from on top of the chest of draws. The member of staff does and apologises. Barbara says it is OK, and that she understands the staff are very busy at the moment. The member of staff says she'll try and remember that Barbara needs glasses to read. She also says that being an agency worker with so few regular staff on duty means that she is having to learn a lot of new information about the residents. For the next hour Barbara reads the magazine, but then returns to not doing a great deal.

Early in the evening the manager of the home comes round to see Barbara. They have a short chat. There seems genuine warmth between them as they speak to one another. Barbara asks about Veronica, Barbara's friend in the home. The manager says that she is fine and hasn't had the virus, but is staying in her room to try and make sure she doesn't catch it. The manager also tells Barbara that she has arranged a video call with her daughter. The manager sets up the call on a tablet that is placed on the bedside cabinet. Once the call starts and it is clear that both parties can see and hear one another, the manager leaves, saying that they'll be back in 15 minutes. Barbara is clearly very happy to see and hear her daughter. The conversation begins with Barbara telling her daughter that she feels much better now, just a little wiped out. The daughter is clearly relieved. Barbara tells her daughter that the staff have been wonderful, but that she is missing talking to people, including Veronica. They like to sit in the same seats in the lounge and natter. They also sit together at mealtimes. Most of all she misses seeing her daughter. The daughter says she will be in once that horrible sickness is all gone. Barbara tells her daughter that feeling sick in front of others was uncomfortable and embarrassing, however, now that she's feeling much better and she hopes she will be able to leave her room tomorrow and go back to the lounge. She is bored of sitting in bed, wants something to do, and someone to talk to. Her daughter says it might not be tomorrow, but it should be soon. Barbara sounds disappointed. The conversation turns to how other family members are. Barbara struggles to remember details about these family members and finds it frustrating. The call ends with Barbara's daughter reminding Barbara that it will soon be back to normal and then she'll be in to see her in person. A few minutes after the call ends, the manager comes in, has a quick chat, and then takes the tablet.

<u>Topic Guide, H&SC Professionals involved in supporting care homes in preventing and managing infectious outbreaks</u>

1. Does this scenario sound like something you have come across before?
    - Which parts of it sound familiar?
    - Is there anything in the scenario that is new or different to what you have come across before?
2. Which parts of Barbara's life do you think may be bothering her most at the moment?
3. Do you have any thoughts about how her carers or family could be doing anything different for her?
4. Do you think there is anything the care home could do in advance to help Barbara, if she gets norovirus again, to help her quality of life whilst she has norovirus and is recovering from it?
5. Do you think that there is anything that the infection control teams locally could support with?
6. We have developed a short questionnaire that helps residents, their families and care homes measure and record different aspects of quality of life in everyday life. The questionnaire (ASCOT) asks about eight different aspects of residents' quality of life, briefly:
    (i) whether residents are happy with the way food and drink is provided
    (ii) their own personal cleanliness,
    (iii) and cleanliness of the home
    (iv) their safety
    (v) how residents spend their time,
    (vi) including time spent with others
    (vii) how much control they have over their lives
    (viii) how they feel about their dignity

    Going back to the scenario, do you think that Barbara's carers have covered all these aspects in helping her with her quality of life during a norovirus outbreak?
    - Do you think this kind of thing would help the agency staff?
    - Do you think this kind of thing would help Barbara and her family?
7. Using this list, has it helped you to think about Barbara's care any differently?
8. Do you think it would help Barbara and her carers if they had a record of her quality of life in everyday life, and how it changed when she went down with norovirus?
9. What do you think staff would think about keeping this kind of record?
10. In your own area of work, considering other kinds of outbreaks that you manage and support, would you find this kind of questionnaire and record helpful in looking after residents' quality of life?
    - Do you think it would have any other benefits?
    - Do you think there would be any down-sides to recording and keeping this kind of information?
11. Is there anything else you think would be helpful in supporting resident's quality of life during an infectious outbreak?
12. Do you have anything else to add, or to ask me?
